# Penetration performance of protective materials from crossbow attack: a preliminary study

**DOI:** 10.1007/s12024-023-00598-2

**Published:** 2023-03-13

**Authors:** James Read, Rachael Hazael, Richard Critchley

**Affiliations:** Defence Academy of the United Kingdom, Cranfield Forensic Institute, Cranfield University, Shrivenham, SN6 8LA UK

**Keywords:** Kinetic energy density, Impact, Polycarbonate, Para-aramid, Ballistic gel, Bolts

## Abstract

Crossbow-related injuries resulting in serious and mortal consequences have increased in recent years, and although significant research exists for both injury and fatality on the human body, limited data exists on the lethality of the bolt and the failure modes of protective materials. This paper concerns itself with the experimental validation of four differing crossbow bolt geometries, their effects on material failure and potentially lethality. During this study, four different types of crossbow bolt geometries were tested against two protection mechanisms that differed in mechanical properties, geometry, mass and size. The results show that at 67 ms^−1^, ogive, field and combo tips do not provide lethal effect at 10-m range, whilst a broadhead tip will perforate both the para-aramid and a reinforced area of polycarbonate material consisting of two 3-mm plates at 63–66 ms^−1^. Although perforation was apparent with a more honed tip geometry, the chain mail layering within the para-aramid protection and friction caused by polycarbonate petalling on the arrow body reduced the velocity enough to demonstrate the materials under test are effective at withstanding crossbow attack. Subsequent calculation of the maximum velocity that arrows could achieve if fired from the crossbow within this study shows results close to the overmatch value of each material and therefore a requirement to advance the knowledge in this field to influence the development of more effective armour protection mechanisms.

**Supplementary Information:**

The online version contains supplementary material available at 10.1007/s12024-023-00598-2.

## Introduction

The number of serious or fatality-related incidents occurring as a resultant of crossbow-related injury has increased in recent years [1–5]. This can be partially attributed to the gap within existing legislation which excludes crossbows from the UK Firearms Act 1968 [6], despite many exceeding kinetic energy density (KED) values shown from air rifles [7] and the ease in which a crossbow can be purchased from both online and high street sources [8, 9]. However, a recent attempted act of terrorism has triggered a review of the current policy by the UK government [10, 11] with the aim to tighten restrictions to better control the sale and use of crossbows.

The combination of the two aforementioned factors has resulted in an increase in criminal activity which requires an active police presence to tackle. Current protection can be categorised into two defined areas: wearable and additional. Current wearable protection is derived from para-aramid materials combining both high strength and carefully tailored stiffness properties [12] which can be achieved by altering the ply orientation [13] to influence high tensile strength characteristics [14].

Such materials can be used in a magnitude of protection applications and are both tested and qualified for their intended use [15, 16]. The crossbow threat can be more closely aligned to the tensile testing element of stab testing due to the readily available tip geometries available [17–20] and velocities being within the sub-ordnance range (79 ms^−1^) [21]; however, it is noted that the impact force generated from a crossbow bolt will differ from that delivered by hand. To date, no work has been undertaken to derive test standards which include the emergent crossbow threats influence on the materials’ ability to distribute stress throughout its matrix [22, 23].

In addition to the wearable protection offered to the user, polycarbonate materials are adopted for use in high-risk riot scenarios or where extra protection is required. This transparent polymer offers a lightweight solution with high ductility and exhibits high yield strain rate responses during perforation [24] when exposed to impact but will depend on both impact conditions and projectile geometry. Extensive qualification is required to ensure polycarbonate materials will provide suitable levels of protection against ballistic threats whilst considering human factor influence [25]. It is noted however that to date no work has been done on the materials’ behaviour when exposed to the crossbow threat but instead captures projectiles with differing geometries and weights within sub-ordnance regimes to record material failure methods against a range of polymers such as polycarbonate.

Previous work has reported that reductions in yield strength and ductility occur when 4-mm-thick polycarbonate samples were subjected to 8.3-g ball bearings travelling at 50 ms^−1^ [26] and further quantified by applying a maximum mechanical stress of 33 MPa to the test samples proving that there was no risk of brittle behaviour. Similar studies have also suggested that impact from more conical-shaped projectiles increased the likelihood of penetration caused by extreme deformation when compared to impacts with flat faces, however, will vary with impact orientation [24]. The previous investigation into polycarbonate’s effectiveness to withstand a pointed projectile suggests that if travelling at greater than 58 ms^−1^, the projectile would penetrate the polycarbonate and increase risk to the user [26].

From the literature, it is evident that whilst this threat continues to grow, the paucity of data of how personal protective equipment (PPE) fairs against such a threat needs to be addressed. This work aims to begin to address those gaps by examining how the most common types of PPE material respond to sub-ordnance impact from varying geometries, therefore establishing how safe the user could remain in crossbow attack scenario. This will be done by assessing the performance of current in-service protective materials against four differing tip geometries fired at three velocities to evaluate the need to provide enhanced levels of protection and to measure any behind armour blunt trauma sustained from impact of the bolts to ensure any risk of injury or fatality is minimised.

## Materials and method

### Crossbow and projectiles

All experiments within this study were conducted using a ‘mid performance’ Jag II crossbow [21] with 3 bow string lengths of 31.5″, 30.5″ and 29.5″ to achieve velocities in the ranges of ~48, ~56 and ~67 ms^−1^ respectively. A composite epoxy bolt was purchased [27] along with four differing steel tip geometries shown in Table 1 [17–20] to adequately capture geometries’ effect on performance. All equipment purchased was deemed representative both technically and value for money of a typical system that a criminal may use.

**Table 1:** Crossbow bolt specification comparison

**Parameter**	**Bolt 1 **[17]	**Bolt 2 **[18]	**Bolt 3 **[19]	**Bolt 4 **[20]
Tip Material	Steel	Steel	Steel	Steel
Tip Shape	Field	Ballistic ‘Ogive’	Combo	Broadhead
Tip Size	8.731mm	8.731mm	8.731mm	10.57mm
Shaft and Fletching Material [27]	Carbon/Fibreglass	Carbon/Fibreglass	Carbon/Fibreglass	Carbon/Fibreglass
Total Mass	25.1g	25.2g	25.0g	25.0g
Nock^a^	Half Moon	Half Moon	Half Moon	Half Moon

^a^The nock is the point at which the arrow aligns to the bow string. It is the point of contact at which maximum force is exerted, allowing the arrow to leave the bow

### Target materials

To ensure a suitable baseline was gathered before testing the protection mechanisms, 10% ballistic gelatine with a bloom number of between 250 and 300 [28] was used to simulate the arrows lethality against an unarmoured target. The material was manufactured, cured and tested in temperature ranges in accordance with the suppliers’ instructions. In addition, the material was successfully calibrated using the Fackler methodology [29]. This material’s suitability for assessment of human torso simulation has been evidenced within numerous studies [36–40]. The gelatine was moulded to provide a depth of 250 mm in accordance with the 50th percentile male principle and included heights and widths of the average male torso [30].

Two test materials were selected from the range of in-service armours available. The wearable protection was of para-aramid construction with an embedded chain mail layer that had been qualified to HG1a/KR1.^1^ This was backed with Roma Plastilina Clay No1 to capture any behind armour blunt trauma as per test specification 039/17 [15] and secured using duct tape. The Roma Plastilina No1 was successfully calibrated in accordance with the HOSDB standard using steel spheres dropped from a height of 1.5 m, showing a mean depth of compression of 15 mm ± 1.5 mm results [31].

The second was a 1200 × 571 × 3 mm Armadillo SH007DS [32] polycarbonate riot shield that was reinforced with a 670 × 430 × 3 mm backing panel located centrally to enable ergonomic handles to be mounted and creating a double skinned area to protect the torso. This was selected as a portable and ‘additional/enhanced’ method of protection. To capture any behind armour effects, the polycarbonate was mounted 300 mm in front of the gelatine block, representative of the average length of the human arm [30] and secured using a weighted block at the bottom of the shield to stop tilting of the target.

### Instrumentation

A Phantom V12 high-speed camera with a resolution of 896 × 400 was set to capture 29,000 frames per second with an exposure of 4 µs and associated PCC software V3.5.792.0 (64 bit) was used to capture the impact event on the tested materials to monitor oscillation, behind armour blunt trauma (BABT) and any potential fragmentation, whilst input and output velocities were captured using a Weibel W700 Radar Doppler.

Where applicable, the use of tape measures to gauge firing heights and handheld rulers to measure indentation and any perforation from the arrows were used. Results were recorded with an error value of ± 0.1 mm.

### Setup

**Fig. 1:**
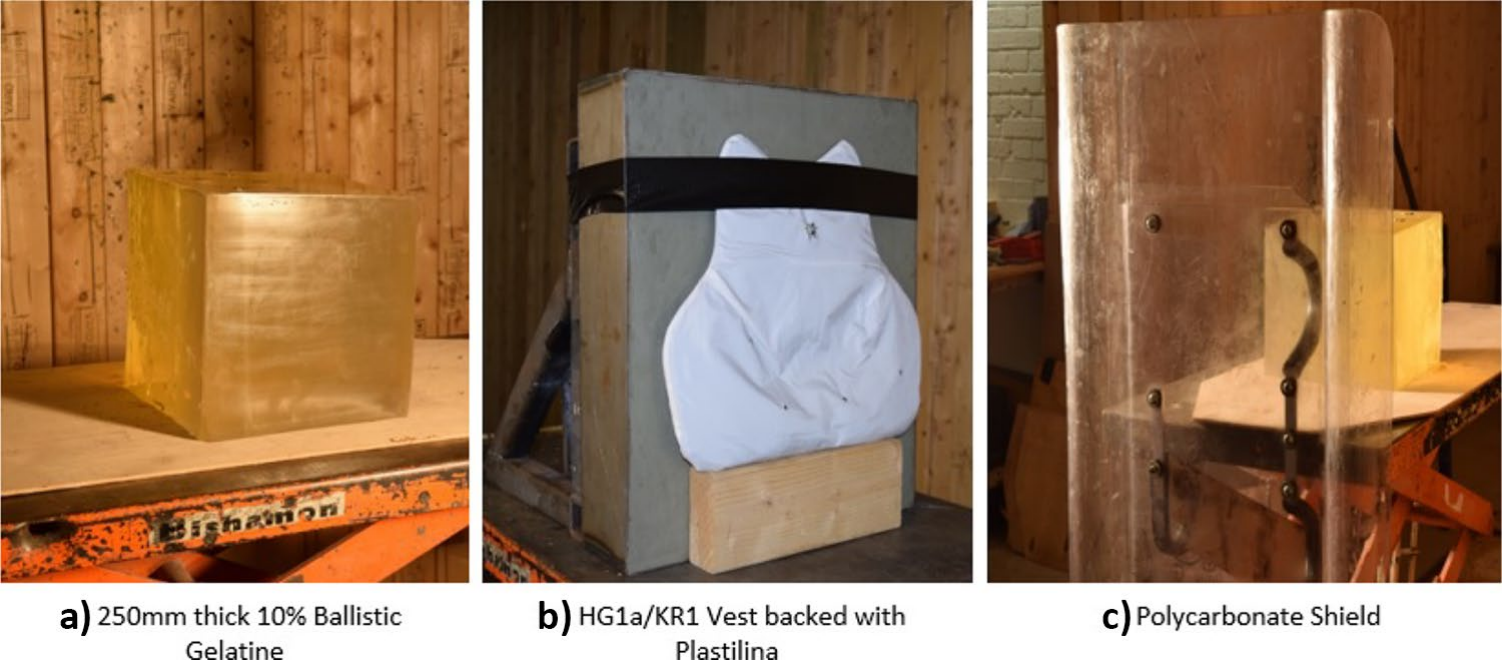
Target material setup showing **a** ballistic gelatine, **b** para-aramid material and **c** polycarbonate work holding arrangements (not to scale)

**Fig. 2:**
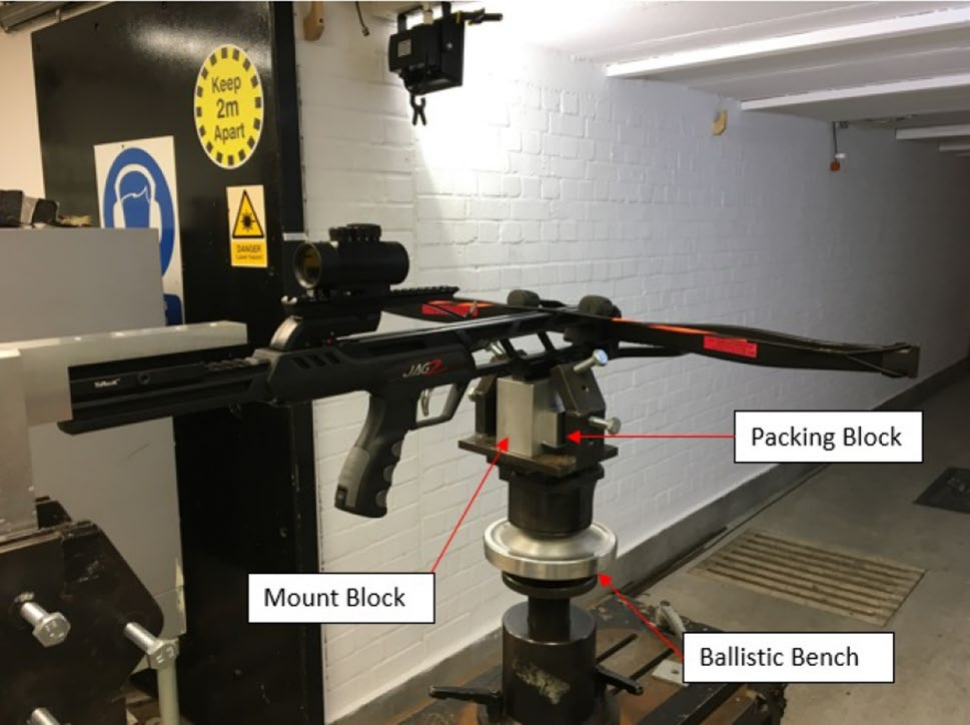
Crossbow mount setup [32]

The test setup adopted for this study was adapted from previous works [34] with minor modifications made (Fig. 1). The crossbow was located on top of a mounting block to hold the crossbow in place, whilst being held central to the ballistic bench jaws with two packing blocks. Both the packing blocks and mounting block were located within a generic firearms mount. The mount encompassed a mechanism to easily adjust the height to coincide with the centre of material mass being tested and line of sight when required to reduce time between testing serials as shown in Fig. 2.

**Fig. 3:**
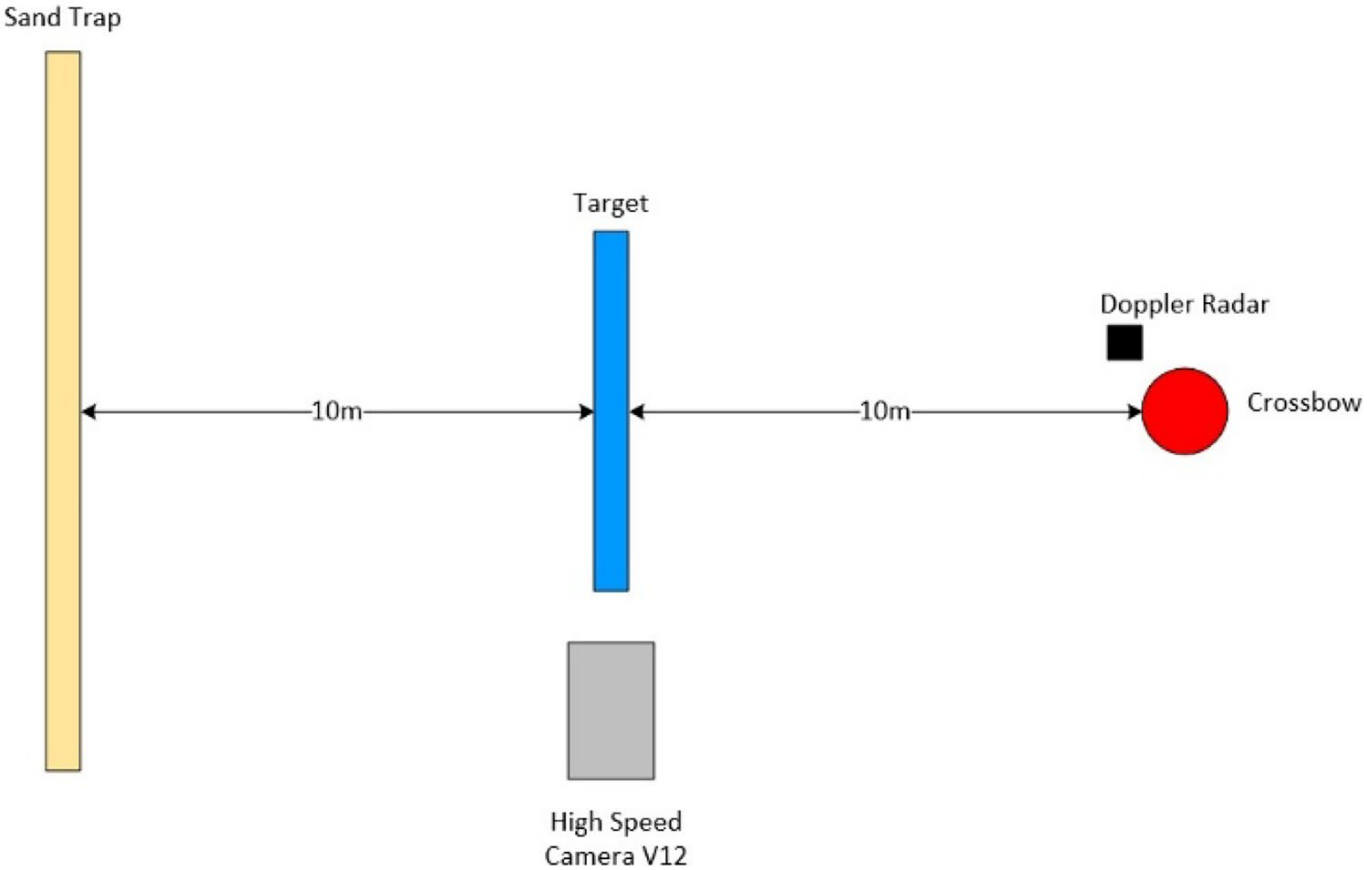
Diagram representation of experimental setup (adapted from [32]). Not to scale

The small arms experimental range (SAER) located at Cranfield Universities Shrivenham Campus was used to conduct this research. The length of flight for the bolt was set to 10 m with a further 10 m used between the test material and a sand trap to ensure adequate arrest should the bolt perforate the material and remain lethal as shown in Fig. 3.

### Test programme details

A total of 69 tests were conducted, where 2 tests of each parameter combination and target material were initially undertaken. To further explore the influence of geometries, influence on lethality, further experimentation was conducted by first conducting 1 test per geometry at 67 ms^−1^ on the para-aramid material to ensure the crossbow was calibrated and similar results shown to those in initial testing, before increasing the number of tests to 3 for the broadhead geometry at the same velocity on para-aramid and both single and double skinned layers of polycarbonate. The complete test matrix is given within Supplementary Information I.

## Results

### Gelatine

Baseline activity on 10% ballistic gelatine was conducted to witness the behaviour of the arrow when penetrating a thickness of 250 mm. All shots used for baselining used bow strings of various lengths to achieve velocities of ~48, ~56 and ~67 ms^−1^ and included 2 repeated firings (*n* = 2) to ensure accuracy of the data was maintained. A repeatability factor of 2 was chosen due to the accuracy of the velocities being recorded during firing. The parameters of such serials can be found at Supplementary Information I. 

At 45 ms^−1^, the penetration depth of all 3 tip types ranged from 230 to 245 mm with no visible damage to the arrow recorded. At 45 ms^−1^, the combo tip exhibited the largest penetration depth. An increase in the velocity resulted in all arrows perforating the material and protruding by 40–90 mm with the combo tip performing most lethally at 56 ms^−1^. Analysis of the arrows post firing showed that no damage had been incurred from the increased velocity. Finally, the velocity was increased to ~67 ms^−1^ with all parameters remaining the same. This resulted the arrow protruding from the rear of the material at distances of between 150 and 175 mm. Interestingly, at the highest velocity the crossbow could achieve, the combo tip produced results that were sub-par to the ogive and field tips which protruded 175 mm and 168 mm respectively.

### Para-aramid

A HG1a/KR1 vest was placed under test. The same ogive, field and combo tip geometries were used to replicate the same testing conducted on the ballistic gelatine material. The velocities during this test were limited to 67 ms^−1^ to measure perforation ability of the arrows and based on results seen during baseline firings. Post-firing analysis revealed no perforation of the vest had occurred; however, the Plastilina backing material showed indentations measuring from 11.9 to 16.3 mm in depth. In all 9 firings (*n* = 3), all tips penetrated the outer jacket of the vest and resulted in small indentations and material being removed from the lower part of the tips from striking the chain mail layer within the armour pack. Furthermore, during shot number 27, the tip became arrested in the chain mail and could not be retrieved.

Three further firings were conducted at 64–65 ms^−1^ using a more aggressive steel broadhead tip which produced the same arrest during contact with the chain mail layer. Due to the geometry of the broadhead, during shots 15–17, perforation occurred creating a puncture within the Plastilina, whilst still exhibiting back face trauma of between 9.7 and 11.8 mm. During visual inspection, it was found that the tip had been damaged from the contact with the chain mail but would remain lethal should it be re-used.

### Polycarbonate material

In all instances, the arrow failed to fully penetrate the material with each arrow exhibiting forms of brittle behaviour on impact. Post-impact damage assessment showed small indentations left on the front face of the polycarbonate material as a result of the tip geometry minimising the impact force and efficiency to penetrate. Further research into why this occurred resulted in a single field tipped arrow being fired at a single skinned area on the same material, which resulted in identical failure modes from the arrow and the same minor witness mark on the material.

To further explore the initial results, a broadhead tip was used on the same arrow shafts and fired using the same parameters to examine how a more aggressive tip geometry could influence the effectiveness of the protective materials. The results show that when using a more aggressive tip geometry, perforation occurs within the single skinned region at distances between 72 and 85 mm and 23 and 27 mm when targeting the double skinned layer of the shield. During firings conducted on the single skin layer, shot 3’s insert became loose, allowing the perforation measurement to increase to 114.3 mm. However, as this was a singular occurrence and is likely to be caused by build quality, this was discounted and a measurement of 85 mm was taken which deducted the insert length to ensure a fair comparison of all shots undertaken during this study. During all firings, review of the high-speed video footage showed significant oscillation during impact of the arrow. This means that a significant shock would be sent into the operators arm and may also result in damage to the polycarbonate microstructure, therefore weakening its integrity and hence its effectiveness.

## Discussion

### Gelatine

**Fig. 4:**
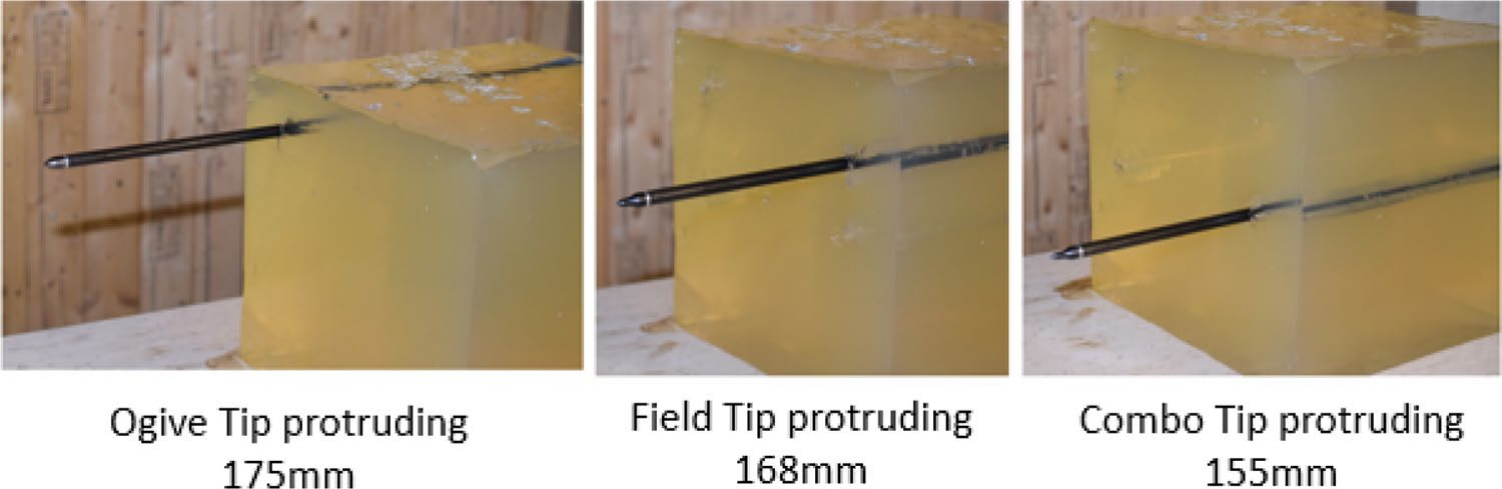
Ballistic gelatine protrusion at 67 ms^−1^

Gelatine was used as a baseline material to measure how differing tip geometries reflect lethality. Figure 4 shows the different bolt behaviours following impact into the gelatine targets. At 47 ms^−1^, the penetration depth of the ballistic, field and combo tips ranged from 230 to 245 mm with the combo tip performing most lethally. Lethality in this paper has been defined as the measure of crossbow bolt ability to penetrate or perforate the desired target. When the velocity was increased to 56 ms^−1^, perforation of the gelatine block was witnessed with all three arrows protruding by 40–90 mm with the combo tip performing most lethally. Finally, when fired at 67 ms^−1^, the bolts protruded between 150 and 175 mm with the ballistic (ogive) tip proving to be marginally more lethal resulting in higher perforation values, and an increase in elastic deformation on impact was witnessed on the front face of the material.

**Fig. 5:**
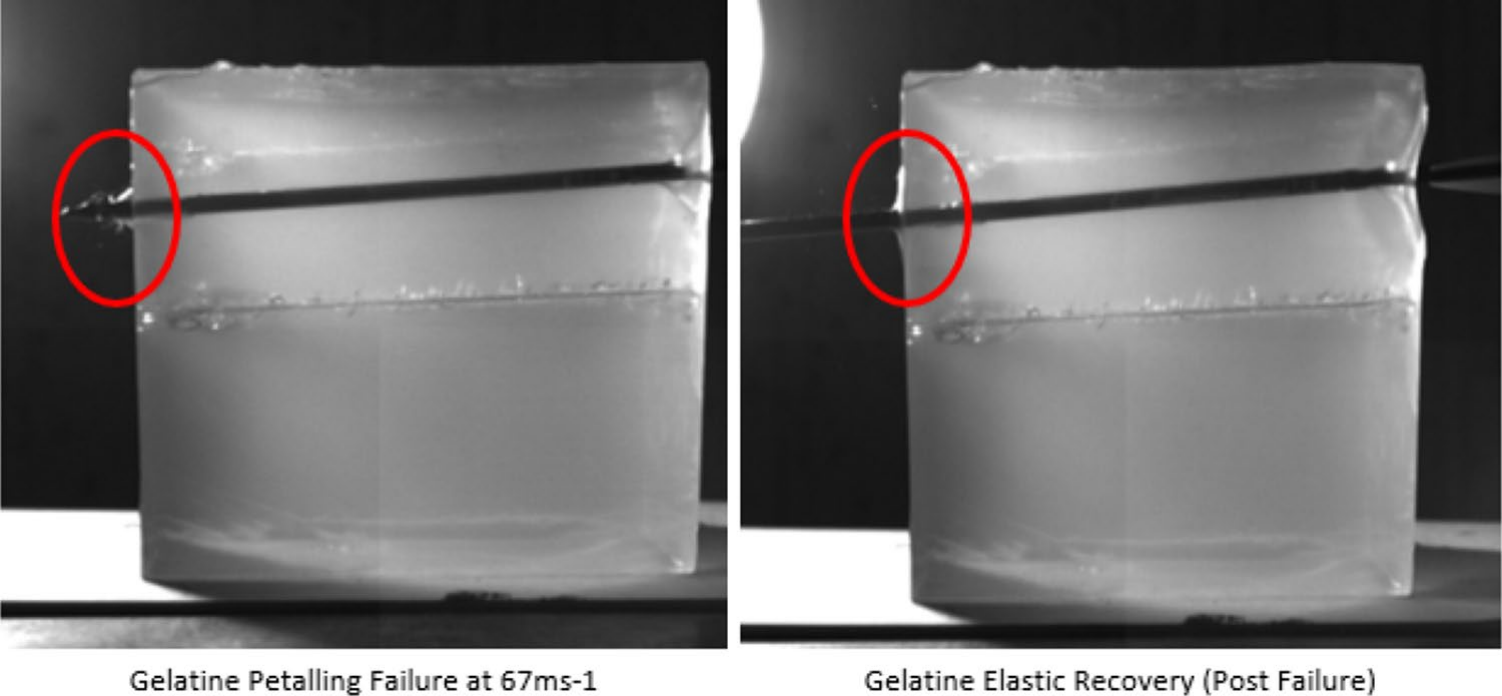
Gelatine petalling and elastic recovery

Furthermore, in all cases, results showed petalling on the back face when perforation was apparent which was later withdrawn as elastic recovery started. This phenomenon has been witnessed within the literature using both experimental and finite element simulation [35] and is shown in Fig. 5. From the results gathered, it is difficult to comment on the survivability of the individual as too many factors play a role in such an event [41–51] and further evidences the need to critically evaluate the protection mechanisms used to defend against this threat.

### Para-aramid

**Fig. 6:**
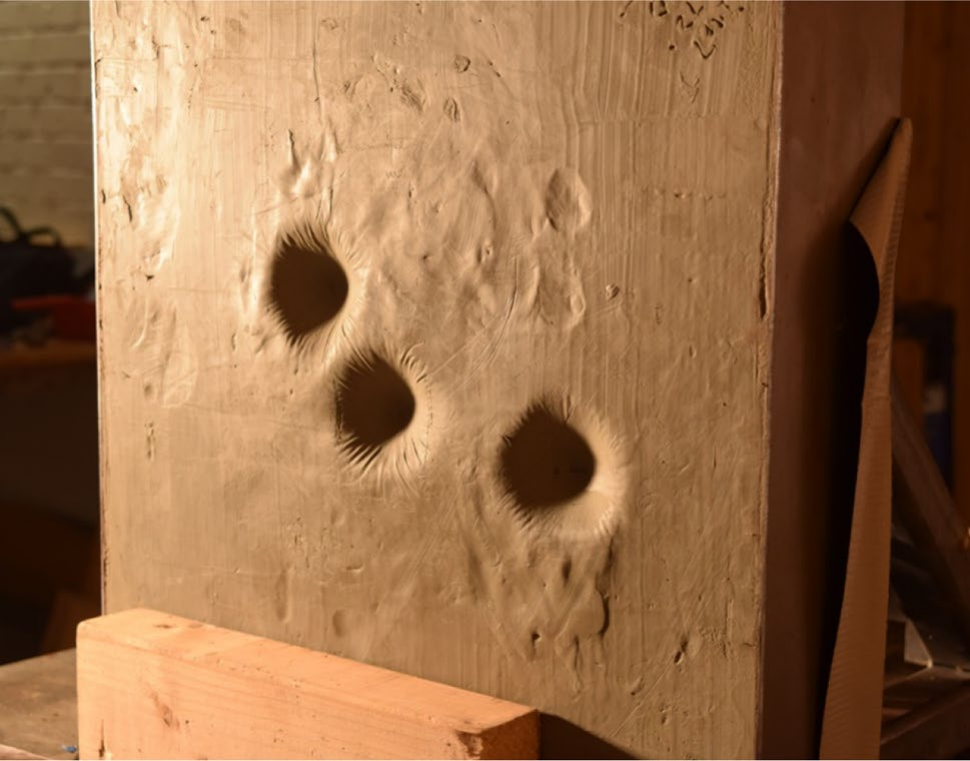
Back face trauma from 67 ms^−1^ impact

Post analysis of the baseline gelatine firings, an assumption was made that for adequate penetration, the crossbow velocity would need to be set at 67 ms^−1^ to achieve sufficient arrest within the material. The ballistic, field and combo tips were fired at the centre mass of the para-aramid material allowing for any yaw from the arrow or aim error to be compensated for. As discussed previously, post-firing visual analysis revealed no perforation had occurred; however, the Plastilina backing material showed conical-shaped indentations measuring from 11.9 to 16.3 mm in depth (Fig. 6) resulting in BABT. The same conical-shaped indentations are witnessed during ballistic testing [52–54] confirming that the shape of BABT is approximately familiar regardless of body armour type [55], whilst depth varies with size and geometry of the projectile and impact velocity. Although this represents distress to the human body, results given within this study are still categorised as ‘acceptable’ within the qualification standards for PPE [31] implying that although rapid deformation of the armour has taken place, it would keep the user safe from serious injury or fatality. The back face trauma measured within this study has been reported to be due to the ability of the material to dissipate energy throughout the interlocking fibres at lower velocities resulting in the material being forced backward which results in greater back face trauma [56].

**Fig. 7:**
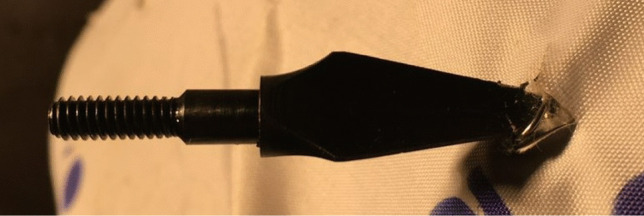
Broadhead arrow tip interaction with chain mail layer

To further explore this phenomenon, a more aggressive tip geometry [20] was fired at 64–65 ms^−1^ to measure the materials ability to withstand a more lethal projectile. The para-aramid material produced the same arrest on impact with the chain mail layer but did result in small indentations and material being removed from the steel tips from striking the chain mail layer within the armour pack. Post-impact analysis revealed similar behind armour trauma from the broadhead tip with the addition of puncture marks caused by the more intrusive geometry being able to fit between the chain mail layers as shown in Fig. 7. Although such behaviour was observed, more repeats are required to determine if this is an outlier result.

**Fig. 8:**
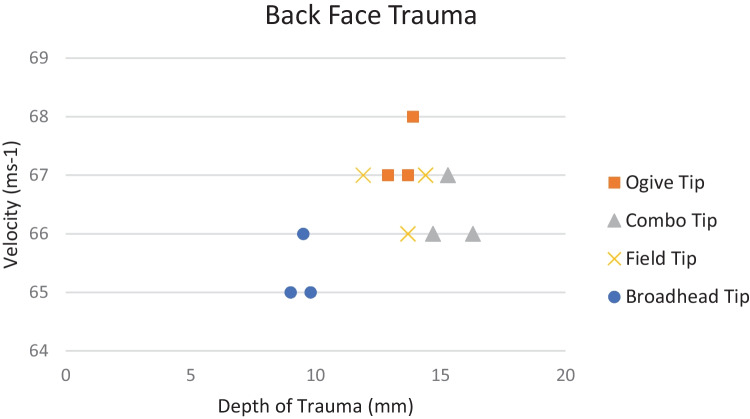
Back face depth of penetration vs impact velocity for tested tip geometries of ogive, combo, field and broadhead

Figure 8 shows that although perforation of the back face of the para-aramid material occurred, it would have not resulted in mortality or severe injury to the user; instead, a similar witness mark with minor bleeding may be apparent should it break through the wearable vest this material is housed inside once worn. This material response has been reported several times [57–59] with the literature concluding that this was caused by multiple influences. Firstly, heat caused by the friction generated when the projectile impacted the sample degraded the fibre performance, enhancing the space between the yarns (response known as trap dooring) [58], but was also caused by both yarn mobility and the projectile geometry [57, 59].

It should be noted that during the works of this study, the maximum velocity that could be achieved was ~67 ms^−1^. This is contrary to the crossbow specification which states a maximum speed of 79 ms^−1^ could be achieved [21]. Assuming that this velocity could be achieved using the arrow configurations presented within this study and fired from close range to minimise energy loss as the arrow leaves the system, the most lethal arrow has been calculated to exhibit a maximum KED of 9 J/mm^2^ as using Eq. (1) and Table 2. As this encroaches on the 10 J/mm^2^ overmatch value for soft ballistic vests [60], there is a potential for failure from influences such as material degradation caused by ageing and wear, therefore leaving the user at risk of penetrating injury.

1 $$\frac{{\frac{1}{2}{\varvec{m}}{\varvec{v}}}^{2}}{{\varvec{A}}}$$

where *m* is the fragment mass (kg), *v* is the impact velocity (ms^−1^) and *A* is the area (mm).

**Table 2:** Maximum achievable KED values

Ogive tip: 8.94 J/mm^2^
Combo tip: 9.00 J/mm^2^
Field tip: 8.97 J/mm^2^
Broadhead tip: 7.38 J/mm^2^

Additionally, using the maximum velocity achieved in this study, Table 3 shows that although yielding values are far lower than the 10 J/mm^2^ overmatch value, material failure and perforation have still been observed. This suggests that the 10 J/mm^2^ value may be for ballistic only events or what the manufacturer quotes for new systems and should be further explored.

**Table 3:** Achievable KED values at 67 ms^−1^

Ogive tip: 6.49 J/mm^2^
Combo tip: 6.44 J/mm^2^
Field tip: 6.47 J/mm^2^

### Polycarbonate

Similar to the firings on the para-aramid material, the velocities for the double skinned polycarbonate experimentation were set to 67 ms^−1^ resulting in the ballistic, field and combo crossbow bolts exhibiting forms of brittle behaviour on impact and subsequently failing to penetrate the material. Post-firing analysis showed indentations left on the front face of the material measuring 6–8 mm; this is predicted to be due to the KED values being insufficient enough to not overmatch the material strength.

Further research into the effect of layering revealed the same results when fired at a single skinned area of the same material using the same tips and resulting in identical failure modes and witness marks. This may have been caused by the differences in mechanical properties of the two materials, in particular the young’s modulus values and the comparison between stress and strain which stop brittle behaviour on impact. Generally, aluminium has proven itself to be more stable in many applications outside of this study and is therefore predicted to be the main influencer of penetrability [61]. To better explore this phenomenon, a field tip geometry was placed onto an aluminium arrow body and was fired at a 3-mm-thick single skinned area of the same polycarbonate material at a velocity of 67 ms^−1^ which exhibited the same bulging effect at the tip insert.

Bulging (swelling of local area due to impact forces acting on weak area, before rapid compression to nominal size) of the arrow at the insert may be due to the ability to match the spine stiffness of the arrow to the performance levels of the crossbow. If the crossbow is of high performance but the arrow shaft material has a lower young’s modulus value, the arrow will be overmatched and fail. The patterns exhibited during testing match that seen at the beginning of compressive failure modes [62]. Additionally, it was found that the diameters of the arrows used in the previous study used an arrow diameter of 8 mm (nominal) compared to the 8.731 mm diameter of this study. This equates to a 20% increase in frontal area for this study, which reduces the KED values and hence reduces lethality which explains the inability to penetrate the polycarbonate. For comparative purposes, a calculation has been conducted using the average mass and maximum velocity parameters of this study to further evidence the influence of arrow diameter on the arrow’s lethality at Table 4.

**Table 4:** KED comparison

Current study: $0.5\times \frac{0.0251 \times {67}^{2}}{8.731}=6.45$ J/mm^2^
Previous study: $0.5\times \frac{0.0252 \times {67}^{2}}{8}=7.04$ J/mm^2^

**Fig. 9:**
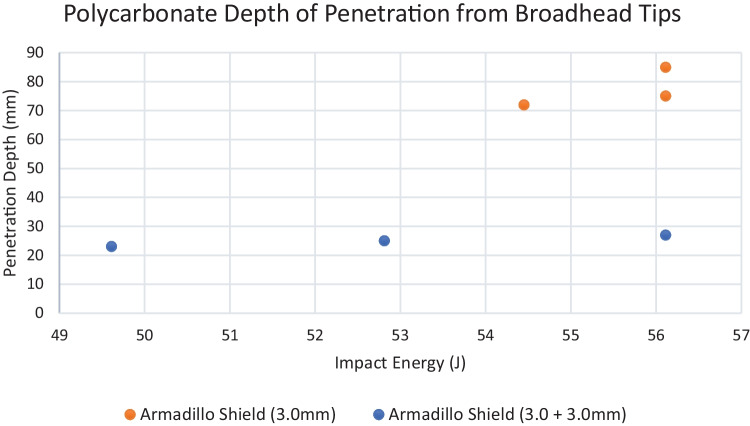
Polycarbonate perforation from broadhead tips

**Fig. 10:**
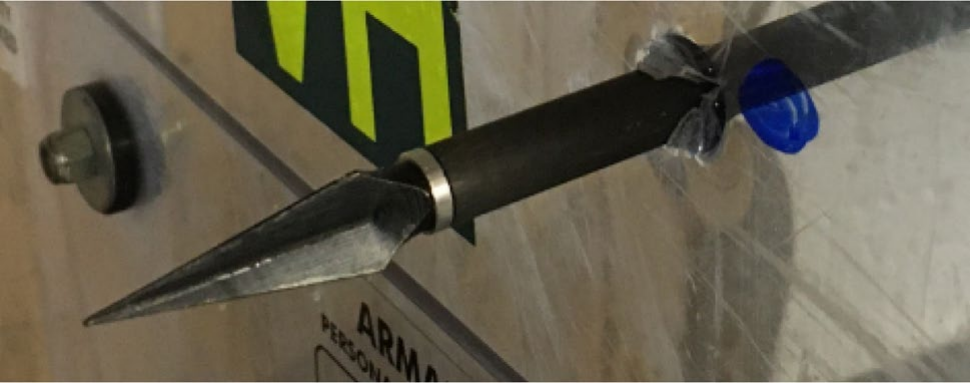
Broadhead tip single skinned area perforation at 67 ms^−1^

**Fig. 11:**
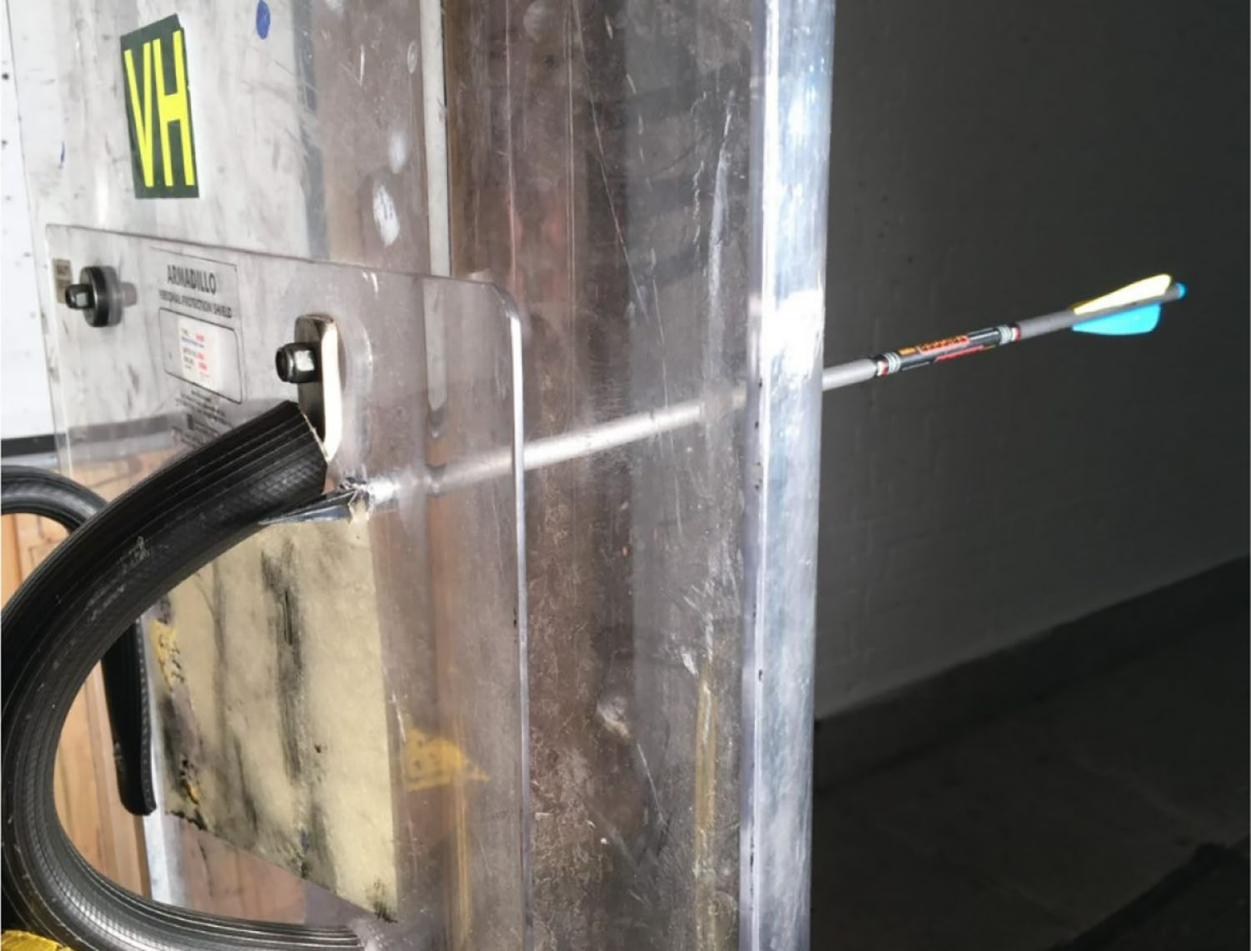
Broadhead tip double skinned area perforation at 67 ms^−1^

Again, a more honed tip geometry was used [21] and fired at the same velocities as the previous firings. The results (Fig. 9) show that when using the broadhead tips, 72–85 mm perforation occurs within the single skinned region and 23–27 mm (Fig. 10) within the double skinned area at the centre (Fig. 11).

However, as the tip size was not significantly bigger than the arrow body, perforation ability would have been diminished by friction caused between the petalling and the arrow body, slowing velocity and therefore reducing perforation. Post-firing analysis of the high-speed video footage showed in all cases significant oscillation from the polycarbonate was present during impact of the arrow representing induced shock to the user’s arm and may have resulted in damage to the polycarbonate microstructure, weakening its integrity and hence its effectiveness at withstanding multiple impacts. This was outside the scope of this study but should be explored further to influence up armouring. Although perforation had occurred, anthropometric measurements [30] show that if operated correctly, the standoff distance between the torso and shield would far exceed the 85-mm perforation presented within this study and would therefore keep the user safe from injury using the parameters within this study. Further work should be done to explore the effects of polycarbonate impact and how responses differ when the polycarbonate is held in different variations.

## Conclusions

A literature review has revealed that limited information exists on personal protective materials’ ability to withstand impacts from crossbow bolts. Furthermore, limited information exists on how lethality is influenced by changing crossbow bolt variables and how the protective materials react when subjected to varying velocities.

Four differing tip geometries have been fired against gelatine, para-aramid and polycarbonate materials at velocities between 48 and 67 ms^−1^ to measure their effectiveness to withstand crossbow attack. Whilst the ogive, field and combo tips exhibited less lethal behaviour under laboratory conditions due to a 20% increase in frontal area, the broadhead tip resulted in significant perforation of the polycarbonate and minor perforation for the para-aramid material.

This work has showed that when exposed to a composite epoxy hybrid arrow with steel tips of differing geometries, the protection mechanisms under test perform advantageously and will keep the user safe from crossbow attack when operated in the correct manner. However, prediction using calculation has shown that at 79 ms^−1^, there is an increased risk of material failure with results showing outputs close to the material overmatch value and further work should be conducted to provide data using more variables.

## Key points

1. HG1A/KR1 Para Aramid Stab Resistant Vest and Polycarbonate Shields perform advantageously when subjected to crossbow arrows with varying tip geometry.
2. Calculations have shown increased risk of material overmatch in velocities exceeding 79 ms^−1^.
3. Increased projectile velocities do not always result in increased lethality.
4. Recommendations to improve protective equipment to meet the needs of the emergent crossbow threat.

### Supplementary Information

Below is the link to the electronic supplementary material.

Supplementary file1 (DOCX 18 KB)

## Data Availability

Raw data was generated at Cranfield University. Both Raw and Derived data supporting the findings of this study are available from the corresponding author on request.

## Footnotes

1 HG1a/KR1 is defined within the 2007 home office body armour standard as how resistant the material is to perforation [33].
