# Supplementary material for: Penetration performance of protective materials from crossbow attack: a preliminary study
Source: Forensic Sci Med Pathol. 2023 Mar 13;20(1):32–42. doi: 10.1007/s12024-023-00598-2 (PMC10944438; doi:10.1007/s12024-023-00598-2)
Supplement: Supplementary file 1 — Supplementary file1 (DOCX 18 KB) [file 12024_2023_598_MOESM1_ESM.docx]

**Supplementary Information I**

| **Crossbow Lethality Test Matrix - Range Day 1** | | | | | | | | | | | | | | | | | | | |  |
| --- | --- | --- | --- | --- | --- | --- | --- | --- | --- | --- | --- | --- | --- | --- | --- | --- | --- | --- | --- | --- |
|  | | | | | | | | | | | | | | | | | | | |  |
| **Trial Reference** | **Project Dates** | | **Bow Type** | | **Bolt Material** | | **Tip Material** | | **Tip Geometry** | **Instrumentation** | **Target Material** | **Distance From Target** | | **Firing Height (From Ground)** | | **Velocities Required** | | **Data Points Required (per Velocity)** | |  |
| Bolt 1_1.1 | 31/03/21 | | Jaguar II | | Carbon/Fibreglass | | Steel | | Field | High Speed Camera, Doppler Radar, PCC Software | Ballistic Gelatine | 10m | | 1.3m | | 47, 56 and 67ms-1 | | 2 | |  |
| Bolt 2_1.1 | 31/03/21 | | Jaguar II | | Carbon/Fibreglass | | Steel | | Ballistic (Ogive) | High Speed Camera, Doppler Radar, PCC Software | Ballistic Gelatine | 10m | | 1.3m | | 47, 56 and 67ms-1 | | 2 | |  |
| Bolt 3_1.1 | 31/03/21 | | Jaguar II | | Carbon/Fibreglass | | Steel | | Combo | High Speed Camera, Doppler Radar, PCC Software | Ballistic Gelatine | 10m | | 1.3m | | 47, 56 and 67ms-1 | | 2 | |  |
|  |  | |  | |  | |  | |  |  |  |  | |  | |  | |  | |  |
| Bolt 1_2.1 | 31/03/21 | | Jaguar II | | Carbon/Fibreglass | | Steel | | Field | High Speed Camera, Doppler Radar, PCC Software | Para-Aramid with Plastilina Backing | 10m | | 1.3m | | 47, 56 and 67ms-1 | | 2 | |  |
| Bolt 2_2.1 | 31/03/21 | | Jaguar II | | Carbon/Fibreglass | | Steel | | Ballistic (Ogive) | High Speed Camera, Doppler Radar, PCC Software | Para-Aramid with Plastilina Backing | 10m | | 1.3m | | 47, 56 and 67ms-1 | | 2 | |  |
| Bolt 3_2.1 | 31/03/21 | | Jaguar II | | Carbon/Fibreglass | | Steel | | Combo | High Speed Camera, Doppler Radar, PCC Software | Para-Aramid with Plastilina Backing | 10m | | 1.3m | | 47, 56 and 67ms-1 | | 2 | |  |
|  |  | |  | |  | |  | |  |  |  |  | |  | |  | |  | |  |
| Bolt 1_3.1 | 31/03/21 | | Jaguar II | | Carbon/Fibreglass | | Steel | | Field | High Speed Camera, Doppler Radar, PCC Software | Polycarbonate | 10m | | 1.3m | | 47, 56 and 67ms-1 | | 2 | |  |
| Bolt 2_3.1 | 31/03/21 | | Jaguar II | | Carbon/Fibreglass | | Steel | | Ballistic (Ogive) | High Speed Camera, Doppler Radar, PCC Software | Polycarbonate | 10m | | 1.3m | | 47, 56 and 67ms-1 | | 2 | |  |
| Bolt 3_3.1 | 31/03/21 | | Jaguar II | | Carbon/Fibreglass | | Steel | | Combo | High Speed Camera, Doppler Radar, PCC Software | Polycarbonate | 10m | | 1.3m | | 47, 56 and 67ms-1 | | 2 | |  |
|  |  | |  | |  | |  | |  |  |  |  | |  | |  | |  | |  |
|  |  | |  | |  | |  | |  |  |  |  | |  | |  | |  | |  |
|  | Variables | |  | |  | |  | |  |  |  |  | |  | |  | |  | |  |
| **Crossbow Lethality Test Matrix - Range Day 2** | | | | | | | | | | | | | | | | | | | |  |
|  |  |  |  |  |  |  |  |  |  |  |  |  |  |  |  |  |  |  |  |  |
| **Trial Reference** | | **Project Dates** | | **Bow Type** | | **Bolt Material** | | **Tip Material** | **Tip Geometry** | **Instrumentation** | **Target Material** | | **Distance From Target** | | **Firing Height (From Ground)** | | **Velocities Required** | | **Data Points Required (per Velocity)** |  |
| Bolt 1_4.1 | | 24/05/2021 | | Jaguar II | | Carbon/Fibreglass | | Steel | Field | High Speed Camera, Doppler Radar, PCC Software | Para-Aramid with Plastilina Backing | | 10m | | 1.3m | | 67ms-1 | | 1 |  |
| Bolt 2_4.1 | | 24/05/2021 | | Jaguar II | | Carbon/Fibreglass | | Steel | Ballistic | High Speed Camera, Doppler Radar, PCC Software | Para-Aramid with Plastilina Backing | | 10m | | 1.3m | | 67ms-1 | | 1 |  |
| Bolt 3_4.1 | | 24/05/2021 | | Jaguar II | | Carbon/Fibreglass | | Steel | Combo | High Speed Camera, Doppler Radar, PCC Software | Para-Aramid with Plastilina Backing | | 10m | | 1.3m | | 67ms-1 | | 1 |  |
| Bolt 4_4.1 | | 24/05/2021 | | Jaguar II | | Carbon/Fibreglass | | Steel | Broadhead | High Speed Camera, Doppler Radar, PCC Software | Para-Aramid with Plastilina Backing | | 10m | | 1.3m | | 67ms-1 | | 3 |  |
|  | |  | |  | |  | |  |  |  |  | |  | |  | |  | |  |  |
| Bolt 1_5.1 | | 24/05/2021 | | Jaguar II | | Carbon/Fibreglass | | Steel | Field | High Speed Camera, Doppler Radar, PCC Software | Polycarbonate | | 10m | | 1.3m | | 67ms-1 | | 1 |  |
| Bolt 2_5.1 | | 24/05/2021 | | Jaguar II | | Carbon/Fibreglass | | Steel | Ballistic | High Speed Camera, Doppler Radar, PCC Software | Polycarbonate | | 10m | | 1.3m | | 67ms-1 | | 1 |  |
| Bolt 3_5.1 | | 24/05/2021 | | Jaguar II | | Carbon/Fibreglass | | Steel | Combo | High Speed Camera, Doppler Radar, PCC Software | Polycarbonate | | 10m | | 1.3m | | 67ms-1 | | 1 |  |
| Bolt 4_5.1 | | 24/05/2021 | | Jaguar II | | Carbon/Fibreglass | | Steel | Broadhead | High Speed Camera, Doppler Radar, PCC Software | Polycarbonate (Single Skin) | | 10m | | 1.3m | | 67ms-1 | | 3 |  |
| Bolt 4_5.2 | | 24/05/2021 | | Jaguar II | | Carbon/Fibreglass | | Steel | Broadhead | High Speed Camera, Doppler Radar, PCC Software | Polycarbonate (Double Skin) | | 10m | | 1.3m | | 67ms-1 | | 3 |  |
|  | |  | |  | |  | |  |  |  |  | |  | |  | |  | |  |  |
|  | |  | |  | |  | |  |  |  |  | |  | |  | |  | |  |  |
|  | | Variables | |  | |  | |  |  |  |  | |  | |  | |  | |  |  |
